# Assessment of water pollution in the Brazilian Pampa biome by means of stress biomarkers in tadpoles of the leaf frog *Phyllomedusa iheringii* (Anura: Hylidae)

**DOI:** 10.7717/peerj.1016

**Published:** 2015-06-04

**Authors:** TG Santos, R Melo, DG Costa-Silva, MEM Nunes, NR Rodrigues, JL Franco

**Affiliations:** 1 Laboratório de Estudos em Biodiversidade Pampeana (LEBIP), Universidade Federal do Pampa, São Gabriel, RS, Brazil; 2 Centro Interdisciplinar de Pesquisas em Biotecnologia (CIPBIOTEC), Universidade Federal do Pampa, São Gabriel, RS, Brazil

**Keywords:** Aquatic contamination, Anuran larvae, Biomarkers, Oxidative stress, Cholinesterase

## Abstract

The Brazilian Pampa biome is currently under constant threat due to increase of agriculture and improper management of urban effluents. Studies with a focus on the assessment of impacts caused by human activities in this biome are scarce. In the present study, we measured stress-related biomarkers in tadpoles of the leaf frog *Phyllomedusa iheringii*, an endemic species to the Pampa biome, and tested its suitability as a bioindicator for the assessment of potential aquatic contamination in selected ponds (S1 and S2) nearby agricultural areas in comparison to a reference site. A significant decrease in acetylcholinesterase activity was observed in S2 when compared to S1 and reference. The levels of total-hydroperoxides were increased in S2 site. In parallel, increased activity of the antioxidant enzymes catalase, superoxide dismutase and glutathione S-transferase were observed in S2 when compared to S1 and reference. Further studies are necessary in order to correlate the changes observed here with different chemical stressors in water, as well as to elucidate mechanisms of toxicity induced by pesticides in amphibian species endemic to the Pampa biome. Nevertheless, our study validates *Phyllomedusa iheringii* as a valuable bioindicator in environmental studies.

## Introduction

The Brazilian Pampa biome, located in the southern Brazil, covers a large grassland territory containing a vast number of endemic species (Bencke, 2009). Currently, this biome has been neglected in terms of environmental protection and conservation of its biodiversity (Roesch et al., 2009). The improper management of urban waste and widespread use of pesticides in monocultures, especially soybeans and rice, are major causes of environmental degradation in this biome (Behling & Pillar, 2007). Up to date studies on risk assessment and biomonitoring are scarce, and the actual impacts of human activities to the Pampa’s environmental quality are poorly understood.

The use of biomarkers in aquatic organisms have been pointed out as an effective approach to obtain information about environmental quality and the potential threats caused by pollutants to the aquatic ecosystem (Viarengo et al., 2007). Biomarkers, by definition, consist in a range of biological responses related to exposure to contaminants and may include molecular, cellular, physiological and behavioral responses (Montserrat, Geracitano & Bianchini, 2003). The measurement of biomarkers at the molecular and cellular levels have been proposed as early hallmarks of exposure to chemical pollutants, thus consisting in reliable and sensitive tools for environmental risk assessment studies (Van der Oost, Beyer & Vermeulen, 2003). For instance, measurements of cholinesterase enzymesand cytochrome P450 (CYP) are considered classical biomarkers, whereas oxidative stress-related parameters, such as antioxidant enzymes and glutathione status are widely used as stress responsive biomarkers (Franco et al., 2010). While cholinesterase enzymes are excellent sensors for aquatic contamination with pesticides including organophosphate and carbamates, CYP proteins are strongly induced during exposure episodes to hazardous organic compounds such as aromatic hydrocarbons (Connon, Geist & Werner, 2012).

Amphibians have a life cycle usually dependent on aquatic and terrestrial ecosystems, highly permeable skin, low mobility, high diversity of reproductive modes, and special physiological requirements, and therefore are often vulnerable to human action (Dunson, Wyman & Corbett, 1992; Tocher, Gascon & Zimmerman, 1997). Despite the vast amount of information regarding biomarkers of aquatic contamination in fish species, little is known about the effects of chemical pollutants on amphibians (Collins & Crump, 2009) and tropical countries as Brazil are no exception (see revision in Kopp et al., 2007). Recently, a marked decline in amphibian populations have been observed and the intensification of habitat loss due to agriculture together with uncontrolled effluent discharges are considered major contributors to the impacts caused by human activities to wild amphibian populations (Collins & Crump, 2009; Orton & Tyler, 2014).

Taking into consideration the scarcity of studies on biomarkers of aquatic pollution within the Pampa biome borders and the limited information about the impacts of chemical pollutants to amphibians, in the present study we aimed to validate the suitability of *Phyllomedusa iheringii* tadpoles for studies on stress-related biomarkers of aquatic pollution. This species is a leaf frog endemic to the Brazilian and Uruguayan Pampa biome whose reproduction is dependent on ponds currently under high agricultural pressure.

## Material and Methods

### Chemicals

5,5-dithio-bis(2-nitrobenzoic)acid (DTNB), 1-chloro-2,4-dinitrobenzene (CDNB), acetylthiocholine iodide, quercetin, *N*,*N*,*N*′,*N*′-Tetramethylethylenediamine (TEMED), 4-(2-hydroxyethyl)-1-piperazine ethanesulfonic acid (HEPES), xylenol orange were purchased from Sigma-Aldrich (Sigma Aldrich, São Paulo, Brazil). All other chemicals were obtained from highest commercial grade available.

## Experimental Animals

In the present study, *Phyllomedusa iheringii* tadpoles were utilized as bioindicators. The subfamily Phyllomedusinae includes 59 species, of which 30 belongs to the *Phyllomedusa* genus (Frost, 2014). *Phyllomedusa* utilizes the vegetation for both vocalization and spawning (Haddad & Prado, 2005; Faivovich et al., 2010). *Phyllomedusa iheringii* Bouleger, 1885 belongs to the *P. burmeisteri* group (*sensu* Lutz 1950) and is a leaf frog endemic to the Uruguayan and Brazilian Pampa, inhabiting forest and grassland ecosystems (Duellman, 1999; Maneyro & Carreira, 2012; Frost, 2014). Reproduction of *P. iheringii* comprises egg deposition into leaves located above the water, where the female folds and glues the leaves, and after about eight days the eggs hatch, releasing exotrophic tadpoles that drop into lentic waters (mode 24 *sensu* Haddad & Prado, 2005) (Langone, 1994). Thus, we chose *P. iheringii* as the model organism because: (1) this species has a prolonged breeding season at the study area, with tadpoles occurring from September to March at the monitored waterbodies (T Santos, 2014, unpublished data); (2) its present high abundance in larval phase, and (3) the larvae are easily sampled and identified.

After being captured, animals were gently transferred to tanks containing water from the capture sites and rapidly transported to the laboratory. No signs of stress or injuries were observed in tadpoles after capturing procedures. Animals were allowed to adapt to the laboratory conditions for at least 3 h under constant aeration and controlled temperature (22 ± 1 °C) before sample preparation. A total of 30 weight and size matched individuals with similar developmental stage (stages 34–36 of Gosner, 1960) was divided in three groups (*n* = 10) and used for the experiments. The experimental groups were: Reference (Ref), Site 1 (S1) and Site 2 (S2). All experimental procedures utilized in this study involving animals were approved by the university’s ethical committee for the use of experimental animals (CEUA Unipampa protocol 043/2013). Field experiments are approved by the Research Council of the Universidade Federal do Pampa (project number: 9.004.13).

### Study area

**Figure 1:**
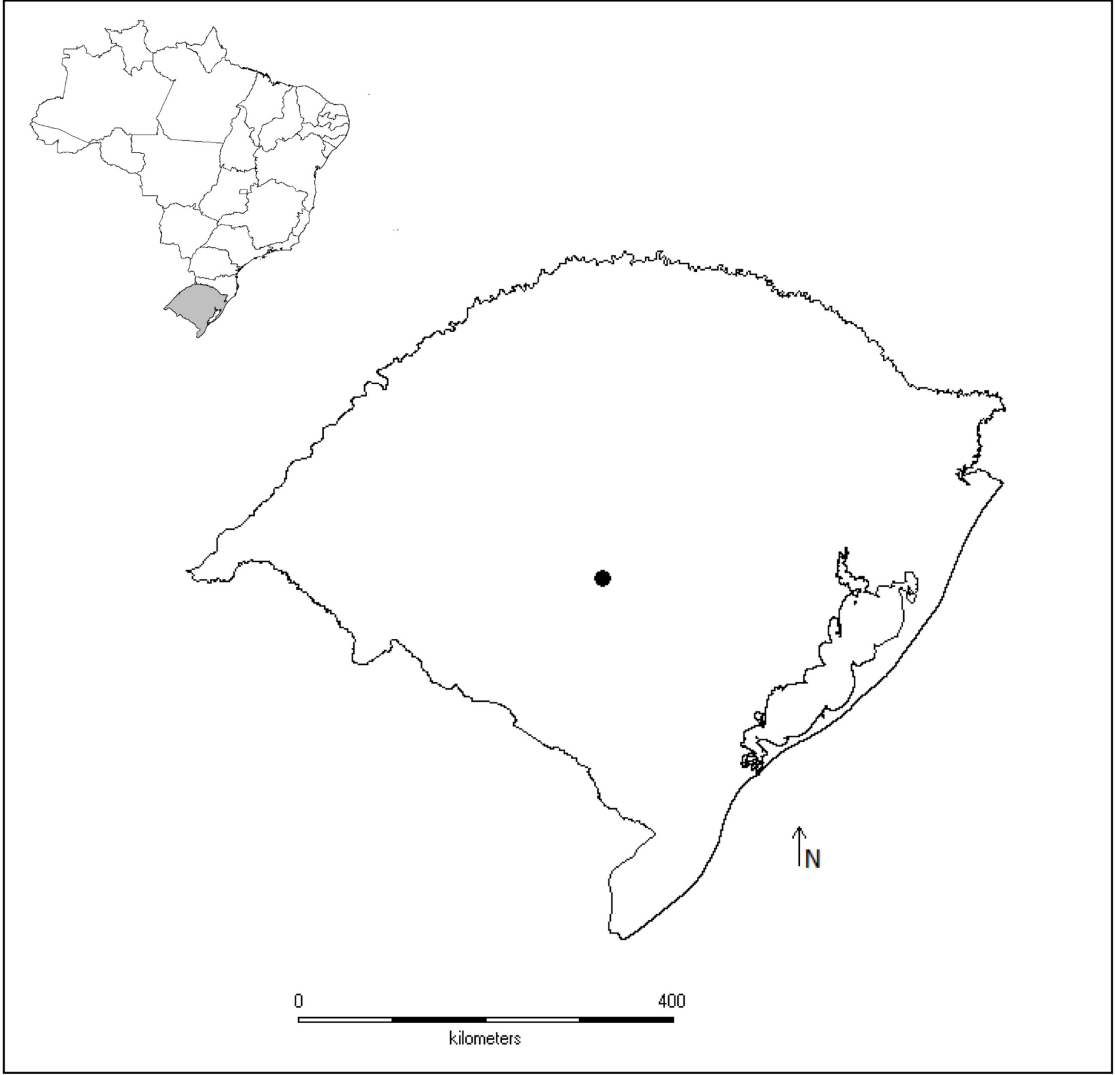
Map of studied sites. Map of Brazil highlighting the geopolitical division Rio Grande do Sul state and the municipality of São Sepé (black point), where tadpoles of the leaf frog *Phyllomedusa iheringii* were studied to access water pollution in the Brazilian Pampa biome by using stress biomarkers.

The study was conducted in two ponds of a private area at the municipality of São Sepé, Rio Grande do Sul state, Brazil (Fig. 1). This area belongs to the Planalto Sul-Rio-Grandense (or Serra do Sudeste), a pampean region characterized by rock crystalline shield outcrops covered by a natural mosaic of grassland (Campos) and seasonal forests (IBGE, 2004). Historically, land use in the region was based on cattle raised on natural vegetation, but this economic activity is now being replaced by soybean (summer season) and wheat (winter season) cultivation. The local climate is classified as subtropical wet (Cfaof Köppen–Geiger’ classification) (Peel, Finlayson & McMahon, 2007), with rainfall evenly distributed throughout the year (1200–1600 mm), i.e., with no dry season (Overbeck et al., 2007). Summer temperatures are high (maximum 40 °C), while winter temperatures are low, with median values less than 15 °C during the three months period when frosts are common. Thus, climatic seasonality is mostly determined by variation in temperature and photoperiod (Both et al., 2008).

The studied three ponds (30°15′03.9″S, 53°35′05.1″W, 198 m; 30°15′25.5″S, 53°34′50.6″W, 216 m), one located in a remnant of natural grasslands (reference site), and two ponds surrounded by soybean and wheat along the year (ponds S1 and S2). Ponds S1 and S2 have been historically used to supply tanks of agricultural pesticides with water and in those occasions the chemical reflux is common.

### Thiol status and total-hydroperoxides

Muscle tissue was collected, weighed and homogenized in 0.5 M perchloric acid (PCA) and centrifuged at 5,000 g for 5 min at 4 °C, and the supernatant was assayed for glutathione levels in the form of non-protein thiols (NPSH). The *pellet* was washed 3 times in 0.5 M PCA and re-suspended in 1 ml 0.1 M TRIS/HCl pH 8.0 for determination of protein thiols (PSH). Both NPSH and PSH were measured spectrophotometrically (Cary 60 UV–Vis; Agilent Technologies) at 412 nm (Ellman, 1959). Data were expressed as µmol NPSH or PSH/g wet tissue.

Total-hydroperoxide levels were evaluated through the xylenol orange assay (Gay & Gebicki, 2002), with minor modifications. In short, frog muscle was homogenized in 20 mM HEPES buffer, pH 7.4 and centrifuged at 1,000 g for 10 min at 4 °C. The supernatant was incubated for 30 min in a reaction medium containing 250 mM perchloric acid, 2.5 mM ammonium iron (II) sulfate hexahydrate, and 1 mM xylenol orange. Hydroperoxide levels were determined at 560 nm using hydrogen peroxide as standard.

### Enzyme activity

For enzymatic analysis, frog muscle was homogenized in 20 mM HEPES buffer, pH 7.4 and centrifuged at 1,000 g for 10 min at 4 °C and an aliquot of supernatant was used for determination of Acetylcholinesterase (AChE). The remained sample was centrifuged at 20,000 g for 30 min at 4 °C for determination of antioxidant enzymes activity. Acetylcholinesterase activity was assayed by measuring the hydrolysis ratio of acetylthiocholine in the presence of DTNB and formation of thionitrobenzoic acid (Ellman et al., 1961), monitored at 412 nm. The glutathione S-transferase (GST) activity was measured as described by Habig & Jakoby, 1981 using 1-chloro-2,4-dinitrobenzene (CDNB) as a substrate. Activity of catalase (CAT) was measured according to Aebi, 1984 and superoxide dismutase (SOD) was measured following the procedures established by Kostyuk & Potapovich, 1989. Data were expressed as mU/mg total protein. Total protein levels were determined according to Bradford (1976).

### Statistical analysis

Statistical analysis was performed using one-way analysis of variance (ANOVA) followed by Dunnett’s *post-hoc* test when needed. Results were considered statistically significant when *p* < 0.05.

## Results

**Figure 2:**
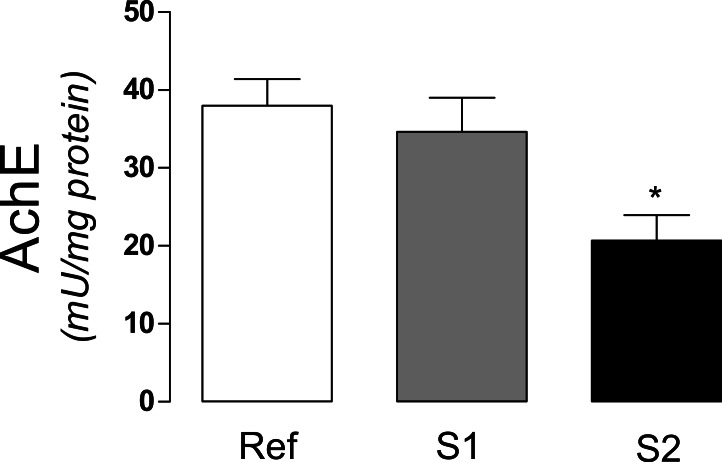
Acetylcholinesterase activity in tadpoles. Acetylcholinesterase activity (AchE) in tadpoles (*Phyllomedusa iheringii*) captured in the Brazilian Pampa biome sites. Data are expressed as Mean ± SD of enzyme activity (mU/mg of total protein). ^∗^*p* < 0.05 when compared to reference site (control).

The activity of acetylcholinesterase (AchE), an extensively used biomarker for organophosphate and carbamate pesticides was significantly decreased (*p* < 0.0163) in S2, when compared to S1 and reference sites (Fig. 2).

**Figure 3:**
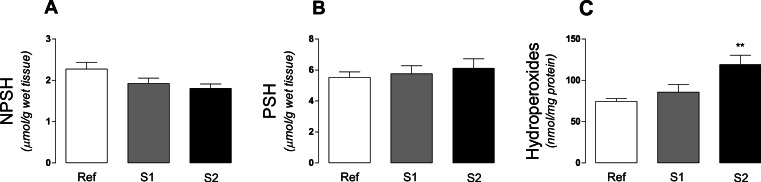
Thiol status and hydroperoxide leves in tadpoles. (A) Non-protein thiols, (B) protein thiols and (C) total-hydroperoxide content in tadpoles (*Phyllomedusa iheringii*) captured in the Brazilian Pampa biome sites. Data are expressed as Mean ± SD of thiol content (µmol/g of wet tissue) and hydroperoxide levels (nmol/mg protein). ^∗^*p* < 0.05 when compared to reference site (control).

We measured thiol status, as non-protein (NPSH) and protein (PSH) thiols and lipid hydroperoxides in order to evaluate a potential oxidative stress condition in tadpoles muscle tissue (Fig. 3). According to the data, a significant (*p* < 0.0044) increase in hydroperoxide levels (Fig. 3A) was observed in S2 site while NPSH and PSH (Figs. 3A and 3B) was not significantly changed. Notwithstanding, a trend (*p* = 0.0588) to decrease NPSH level in S2 was observed.

**Figure 4:**
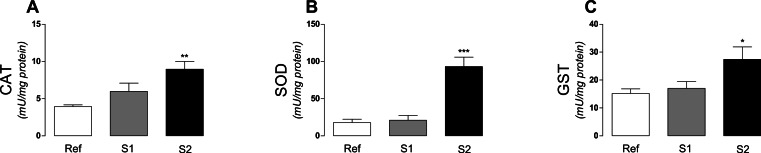
Antioxidant enzymes in tadpoles. Enzymatic activity of (A) CAT, (B) SOD and (C) GST in tadpoles (*Phyllomedusa iheringii*) captured in the Brazilian Pampa biome sites. Data are expressed as Mean ± SD of enzyme activity (mU/mg of total protein). ^∗^*p* < 0.05, ^∗∗^*p* < 0.01 and ^∗∗∗^*p* < 0.001 when compared to reference site (control).

Regarding the activity of antioxidant enzymes, a significant (*p* < 0.0028) increase of catalase (CAT) activity was observed at S2 (Fig. 4A). Superoxide dismutase (SOD) activity was also substantially enhanced (*p* < 0.001) at S2 when compared to S1 and reference (Fig. 4B). The glutathione S-transferase (GST) activity was significantly (*p* < 0.00239) increased in the muscle of tadpoles captured at S2 site, when compared to both S1 and reference (Fig. 4C).

## Discussion

Up to date, little is known about the adverse influences of human activities on the overall environmental quality of the Pampa biome. Studies on biomarkers of water contamination are limited, and the development of suitable risk assessment protocols have been neglected for decades. In the present study, by measuring changes well established biomarkers of water pollution, we validated the use of *Phyllomedusa iheringii* tadpoles, an endemic frog species to the Pampa biome, as a valuable tool for the evaluation of harms caused by human activities to aquatic ecosystems and wild life equilibrium. By observing changes in classical biomarkers, such as acetylcholinesterase activity along with xenobiotics/oxidative stress related parameters in tadpoles sampled in ponds with historical records of agricultural use, we drawn attention for the potential presence of pesticides at harmful levels at the studied area.

Pesticides have widespread application and are believed to have relative benign effects on non-target species, but these risks are often not studied at relevant ecological, spatial or temporal scales (Boone & Bridges, 2003). Acetylcholinesterase (AChE) is a classic biomarker for the presence of sublethal concentrations of organophosphorous and carbamate compounds, which are widely used for pest control and can reach water streams through agricultural and urban releases (Viarengo et al., 2007). Several studies using fish species have shown inhibition of cholinesterase activity in the presence of organophosphate compounds in water (Sancho, Ferrando & Andreu, 1997; Dutta & Arends, 2003). In addition to organophosphate and carbamates, other agricultural compounds such as glyphosate are shown to inhibit AchE in fish (Moraes et al., 2007). Regarding organophosphate compounds in frogs, recent studies have shown the accumulation of such compounds in amphibians (Kittusamy et al., 2014). Glyphosate is also shown to inhibit AchE in frogs (Ruamthum et al., 2011). Even though the effects of AchE inhibitors have been demonstrated in amphibian experimental models *in vitro* and *in vivo* (Gungordu & Uckun, 2014), few studies have been undertaken in order to address the impacts of such pollutants *in situ*, especially to wild frog species. In the present study, we found a significant decrease in the activity of AchE in tadpoles captured in ponds utilized for crop activities in the Brazilian Pampa biome. The two ponds (S1 and S2) in which tadpoles were captured are constantly used for farm irrigation purposes, a fact that potentially increases the probability of pesticide releases into the water. The observed decrease in AchE activity in tadpole muscle tissue may reflect the presence of cholinesterase inhibitors at harmful levels, mainly at the S2 site. In line with this hypothesis, Table 1 shows the most utilized agrochemicals (in descending order) used in the Rio Grande do Sul state, according to Brazilian regulatory agencies (Barreto, Herman & Garibotti, 2012). Among them, four compounds are AchE inhibitors, as organophosphates (acephate and methamidophos), glycine analogs (glyphosate) and carbamates (carbofuran). Although measurements of pesticides concentrations in the selected ponds were not undertaken here, the historical records of land use may suggest that one or more of such compounds may be responsible for the inhibitory effect towards AchE in tadpoles of *Phyllomedusa iheringii*.

**Table 1:** Agrochemicals. List of most commonly used agrochemicals in Rio Grande do Sul State, Brazil (Adapted from Barreto, Herman & Garibotti, 2012).

*Agrochemical*	*Chemical group*
Glyphosate	Glycine analogue
Acephate	Organophosphate
Difenoconazole	Triazole
Methamidophos	Organophosphate
Metalaxyl	Phenylamide
Cypermethrin	Pyrethroid
Diflubenzuron	Benzamide
Carbofuran	Carbamate

Oxidative stress, which is defined as an unbalance between pro- and antioxidants in organisms (Halliwell & Gutteridge, 2007) has been shown as an important mechanism of toxicity of environmental contaminants, including AchE inhibitors (Karami-Mohajeri & Abdollahi, 2011; Lushchak, 2011; Hellou, Ross & Moon, 2012). The presence of persistent organic pollutants in water may result in induction of reactive oxygen species (ROS) and consequently, oxidative stress in aquatic organisms (Lushchak, 2011; Hellou, Ross & Moon, 2012). Then, observing changes in oxidative stress-related parameters and use them as biomarkers of exposure to contaminants in aquatic of semi-aquatic animals may represent a valuable tool for the assessment of environmental quality.

Under oxidative stress conditions, a cellular adaptive response may take place in order to counteract the deleterious effects of oxidative stress (Lushchak, 2011; Schulke et al., 2012). The adaptive response to oxidative challenges is mediated by the transcription factor Nrf2 through the antioxidant response element (ARE). Once oxidative stress signals are generated, Nrf2 triggers the transcription of endogenous antioxidant enzymes as glutathione S-transferase (GST), glutathione peroxidase (GPx), glutathione reductase (GR), superoxide dismutase (SOD), catalase (CAT), NAD(P)H oxidase, NADPH quinine oxidoreductase (NQO-1), glutamate-cysteine ligase (GCL) and thioredoxin system through its binding to the DNA sites known as the antioxidant responsive element “ARE” (Lushchak, 2011; Schulke et al., 2012). We observed a significant increase in the activity of CAT, SOD and GST in tadpoles captured at S2 site (Fig. 4). GST activity is related to detoxification of xenobiotic compounds being widely used as a biomarker for environmental exposures to exogenous compounds (Stegeman & Lech, 1991; Hellou, Ross & Moon, 2012). The increase in GST activity can be interpreted as an adaptive response in order to eliminate toxic xenobiotics present in water and potentially accumulating in tadpoles muscle tissue. The increase in CAT and SOD, which are primary enzymes in the control of intracellular ROS levels (Halliwell & Gutteridge, 2007) may also be interpreted as an adaptive response in tadpoles to survive under the presence of oxidative stressors, corroborating previous literature reports (Clasen et al., 2012).

The activity of antioxidant enzymes, the amount of thiol groups and markers of ROS-induced damage to biomolecules are frequently used for monitoring the presence of pro-oxidant compounds in aquatic environments (Trevisan et al., 2013). The levels of thiols, both in proteins (PSH) or low molecular weight compounds (NPSH), is an indicative of the antioxidant capacity of the organism (Reischl et al., 2007). Markers of oxidative damage to biomolecules, such as lipid peroxidation by-products can be environmentally induced in aquatic organisms (Lushchak, 2011). We observed a significant increase in lipid hydroperoxides and a trend to decrease NPSH levels. NPSH represents an index of cellular levels of glutathione (GSH), a key low-molecular ROS scavenger in living organisms (Ellman, 1959; Lushchak, 2011). Together, these results are indicative of a pro-oxidative condition in which tadpoles are exposed in the studied sites. Pesticides that inhibit AchE can influence amphibian by direct and/or indirect effects, such as paralysis, reduction of foraging, increase predation rates, as well as prohibit or delay metamorphosis and cause death (see review in Boone & Bridges, 2003). In this way, we suggest that future studies should address how chemical stressors interact with abiotic (temperature, pH, ultraviolet light) and biotic (competition and predation) environmental factors, and to measure possible effects of these contaminants on population juveniles recruitment.

Overall, by using tadpoles as bioindicators we observed changes in biomarkers related to the presence of cholinesterase inhibitors and oxidative stress inducers in the studied sites. Since amphibians are especially prone to the adverse effects of water contaminants, further studies are necessary in order to correlate the changes observed here with different chemical stressors in water, as well as to elucidate mechanisms of toxicity induced by pesticides in amphibian species endemic to the Pampa biome. Nevertheless, our study validates *Phyllomedusa iheringii* as a valuable bioindicator in environmental studies.

## Supplemental Information

10.7717/peerj.1016/supp-1 Supplemental Information 1 Raw data

## Additional Information and Declarations

### Field Study Permissions

The following information was supplied relating to field study approvals (i.e., approving body and any reference numbers): Field experiments are approved by the Research Council of the Universidade Federal do Pampa (project number: 9.004.13).
