# Supplementary material for: Assessment of water pollution in the Brazilian Pampa biome by means of stress biomarkers in tadpoles of the leaf frog Phyllomedusa iheringii (Anura: Hylidae)
Source: PeerJ. 2015 Jun 4;3:e1016. doi: 10.7717/peerj.1016 (PMC4458136; doi:10.7717/peerj.1016)
Supplement: Supplemental Information 1 [file peerj-03-1016-s001.docx]

| **Santos et al 2014** | |  |  |
| --- | --- | --- | --- |
|  |  |  |  |
| **Raw data** |  |  |  |
|  |  |  |  |
| Catalase Activity (CAT) | | |  |
|  |  |  |  |
|  | REF | S1 | S2 |
| 1 | 3.76 | 2.29 | 9.89 |
| 2 | 3.89 | 2.12 | 11.72 |
| 3 | 3.68 | 6.30 | 10.56 |
| 4 | 4.30 | 6.83 | 13.86 |
| 5 | 3.70 | 5.35 | 5.91 |
| 6 | 3.61 | 7.12 | 7.53 |
| 7 | 3.07 | 5.50 | 6.34 |
| 8 | 5.41 | 12.24 | 5.86 |
| **mean** | **3.93** | **5.97** | **8.96** |
| **SD** | **0.69** | **3.17** | **3.00** |
| **n** | **8.00** | **8.00** | **8.00** |
|  |  |  |  |
|  |  |  |  |
| Superoxie activity (SOD) | | |  |
|  | REF | S1 | S2 |
| 1 | 17.52 | 24.44 | 21.74 |
| 2 | 4.93 | 6.59 | 116.12 |
| 3 | 22.32 | 19.30 | 90.44 |
| 4 | 43.55 | 63.38 | 134.17 |
| 5 | 26.28 | 22.78 | 63.10 |
| 6 | 11.85 | 14.76 | 107.11 |
| 7 | 6.25 | 7.65 | 118.13 |
| 8 | 10.30 | 8.48 | 93.72 |
| **mean** | **17.87** | **20.92** | **93.06** |
| **SD** | **12.79** | **18.50** | **35.93** |
| **n** | **8.00** | **8.00** | **8.00** |
|  |  |  |  |
|  |  |  |  |
| Glutathione s-transferase activity (GST) | | | |
|  | REF | S1 | S2 |
| 1 | 18.02 | 17.22 | 21.41 |
| 2 | 11.84 | 12.96 | 53.63 |
| 3 | 10.27 | 19.76 | 22.74 |
| 4 | 20.53 | 31.81 | 25.20 |
| 5 | 11.06 | 14.97 | 15.96 |
| 6 | 21.88 | 12.91 | 25.62 |
| 7 | 11.27 | 9.10 | 38.57 |
| 8 | 16.61 | 17.50 | 15.92 |
| **mean** | **15.19** | **17.03** | **27.38** |
| **SD** | **4.65** | **6.83** | **12.77** |
| **n** | **8.00** | **8.00** | **8.00** |
|  |  |  |  |
|  |  |  |  |
| Acetylcholinesterase activity (AchE) | | | |
|  | REF | S1 | S2 |
| 1 | 52.69 | 23.03 |  |
| 2 | 36.25 | 62.14 | 33.64 |
| 3 | 29.93 | 33.01 | 18.43 |
| 4 | 50.01 | 39.45 | 27.22 |
| 5 | 41.08 | 25.75 | 15.90 |
| 6 | 24.11 | 33.51 | 16.05 |
| 7 | 32.44 | 33.85 | 12.79 |
| 8 | 37.22 | 26.55 |  |
| **mean** | **37.97** | **34.66** | **20.67** |
| **SD** | **9.72** | **12.32** | **8.03** |
| **n** | **8.00** | **8.00** | **6.00** |
|  |  |  |  |
|  |  |  |  |
| Non-protein thiols (NPSH) | | |  |
|  | REF | S1 | S2 |
| 1 | 2.89 | 1.36 | 1.64 |
| 2 | 2.57 | 2.00 | 2.22 |
| 3 | 2.67 | 2.34 | 1.46 |
| 4 | 1.52 | 1.97 | 2.24 |
| 5 | 1.91 | 1.82 | 1.93 |
| 6 | 2.22 | 2.44 | 1.61 |
| 7 | 1.97 | 1.99 | 1.51 |
| 8 | 2.46 | 1.47 | 1.84 |
| **mean** | **2.28** | **1.92** | **1.81** |
| **SD** | **0.46** | **0.38** | **0.30** |
| **n** | **8.00** | **8.00** | **8.00** |
|  |  |  |  |
|  |  |  |  |
| Protein thiols (PSH) | |  |  |
|  | REF | S1 | S2 |
| 1 | 5.08 | 4.58 | 8.98 |
| 2 | 6.33 | 5.22 | 3.66 |
| 3 | 6.57 | 7.56 | 6.76 |
| 4 | 5.42 | 6.89 | 5.61 |
| 5 | 6.44 | 7.04 | 6.00 |
| 6 | 4.72 | 3.66 | 7.31 |
| 7 | 3.62 | 4.41 | 4.06 |
| 8 | 5.99 | 6.69 | 6.55 |
| **mean** | **5.53** | **5.76** | **6.11** |
| **SD** | **1.02** | **1.46** | **1.72** |
| **n** | **8.00** | **8.00** | **8.00** |
|  |  |  |  |
|  |  |  |  |
| Total hydroperoxides | | |  |
|  | REF | S1 | S2 |
| 1 | 89.11 | 89.45 | 93.40 |
| 2 | 72.68 | 98.57 | 70.07 |
| 3 | 66.36 | 67.44 | 94.57 |
| 4 | 86.44 | 75.88 | 103.65 |
| 5 | 77.50 | 102.18 | 152.33 |
| 6 | 60.53 | 79.93 | 152.48 |
| 7 | 68.87 | 130.28 | 149.22 |
| 8 | 73.65 | 42.98 | 136.43 |
| **mean** | **74.39** | **85.84** | **119.02** |
| **SD** | **9.72** | **25.98** | **32.36** |
| **n** | **8.00** | **8.00** | **8.00** |
|  |  |  |  |
|  |  |  |  |
| Enzyme activity data are expressed as Mean±SD of enzyme activity (mU/mg of total protein). | | | |
| Thiols data are expressed as Mean±SD of thiol content (micromol/g of wet tissue) | | | |
| Hydroperoxide levels are expressed as Mean±SD (nmol/mg protein) | | | |

REF: reference site

S1: site one

S2: site two
